# Microstructure and radiation shielding capabilities of Al-Cu and Al-Mn alloys

**DOI:** 10.1038/s41598-024-76177-4

**Published:** 2024-11-05

**Authors:** Moamen G. El-Samrah, Islam M. Nabil, Mohamed E. Shamekh, M. Elmasry, M. Osman

**Affiliations:** 1 Nuclear Engineering Department, Military Technical College, Kobry El-kobbah, Cairo, Egypt; 2 Physics Department, Faculty of Science, Fayoum University, Fayoum, Egypt; 3 Material Science and Technology Department, Military Technical College, Kobry El-kobbah, Cairo, Egypt

**Keywords:** Aluminum-copper type-2024 alloy, Aluminum-manganese type-3003 alloy, Radiation shielding, MCNP5, Phy-X/PSD, MRCsC, Physics, Materials science, Condensed-matter physics

## Abstract

In this study, the microstructure and elemental analysis of aluminum-copper alloy type-2024, Al-2024, and aluminum-manganese alloy type-3003, Al-3003, have been investigated by using a scanning electron microscope (SEM) equipped with Energy dispersive spectroscopy (EDS) detector. Experimental and theoretical radiation shielding studies were performed to assess the radiation shielding capabilities of the studied alloys. Considering the radiation shielding theoretical assessment, some reliable software tools were used, such as Phy-X/PSD, MCNP5, NXCom, and MRCsC. The microstructural observations and results have shown the presence of second phases rich with the main alloying elements in both alloys. Considering Al-2024 alloy, coarse second-phase particles, having a size range of 8–15 μm, were found aligning in lines parallel to the rolling direction, whereas smaller ones, having a size range of 2–8 μm, were found decorated the grain boundaries. Also, dark holes represent the pull-out large particles separated during preparation indicated poor adhesion with the main matrix that could be a result of losing particle coherency with the matrix where the misorientation in-between the atomic planes increase. However, better adhesion of the second-phase particles with the matrix, which were found possessing smaller particle size, have been observed in the Al-3003 alloy indicating good coherency and better manufacturing process for the non-heat-treatable alloy. The second-phase particles in case of Al-2024 alloy were found containing significant content of high-Z elements like Cu with greater volume fraction equals 7.5%. On the other side, Al-3003 alloy has possessed second-phase particles which lack of high-Z elements with only volume fraction equals 3.5%. All the former besides the higher density and content of high-Z elements like copper in Al-2024 alloy in compare to Al-3003 alloy and pure aluminum, led to relatively better radiation shielding capabilities against energetic photons, the highest in the low energy band and decreases with the increase of the photon energy, and slight superiority in the case of fast neutrons with only 3%inc. over pure aluminum. For instance, the radiation protection efficiency (RPE) values dropped from about; 23.2, 21.6, and 20.8% at 0.100 MeV to only 5.7, 5.9, and 5.6% at E_γ_ = 2 MeV, for; Al-2024, Al-3003, and Al-Pure, respectively."Please check and confirm that the authors and their respective affiliations have been correctly identified and amend if necessary.""confirmed"

## Introduction

Because of their low weight, robust resistance to corrosion, high specific strengths, and rigidity, aluminum alloys find widespread application in the aerospace industry, the manufacturing of aircraft components, the automobile industry, and the electronic device industry. Because of the way in which they are processed, these alloys can be divided into two categories: wrought and cast. Furthermore, within each category, there are subgroups that are heat-treatable and non-heat-treatable, which are determined by the mechanisms that are responsible for strengthening the alloy^1^.

The heat-treatable aluminum alloy type-2024, reported in this investigation, has attractive characteristics, including high specific strength, good fracture toughness, and excellent fatigue properties with no significant drop in elasticity during the strengthening treatment^2,3^. These pronounced properties make this alloy among the first candidates in aerospace and other critical fields^4,5^. The presence of copper and magnesium in this alloy enhances the formation of coherent dispersoids that strongly hinder the dislocation motion during deformation and significantly raise its strength^6^.

Low density, excellent plasticity, formability, corrosion resistance, and weldability are some of the characteristics that are exhibited by the non-heat-treatable aluminum alloy type 3003. These properties have garnered an increasing amount of attention in a variety of fields, particularly those that require superior formability in order to obtain components with complex shapes^7–9^. The strength of this alloy depends on the cold working mechanism rather than heat treatment. The preliminary grain size of the deformed alloy and the induced dislocation density affect the strengthening level^10,11^. Manganese addition encourages the formation of Al_6_Mn dispersoids that accumulatively build up dislocation density by the Frank-Read mechanism and block slip during deformation. As a result, the yield and ultimate strengths of the alloy are increased without sacrificing its flexibility but retaining its formability^12^.

The use of aluminum alloys, especially Al alloy type-2024 and also Al alloy type-3003 in parts that possess complex geometrical shapes, in aviation and aerospace applications such as space shuttles and passenger aircrafts structural parts and bodies manufacturing, and considering the significant background radiation fields and cosmic rays when flying at high altitudes or living in space, especially for long periods, all that raise the importance of investigating the radiation shielding properties of these alloys and provide the sufficient motivation to conduct this study^13,14^.

Speaking on the formerly denoted importance, researchers have conducted studies to assess radiation shielding properties of some widely used aluminum alloys, knowing that radiation shielding studies on alloys are not comparable to the number of studies in the same field concerning concretes^15–18^, building materials^19–21^, composites^22–24^, and glass systems^25,26^.

Aluminum alloys such as Al-Li, Duralumin, Hydronalium, Italma, Magnalium, Ni–Ti-Al, Y-alloy, and Al_25_Zn Alloy have been investigated as possible shields against ionizing radiation^27–30^. Ni-Ti–Al aluminum alloy and Al_25_Zn Alloy, when adding a permissible percent of titanium, were found to have proper radiation shielding properties, specifically considering X-rays and low energy γ-rays^28,29^. On the other hand, Al-Li alloy was effective in attenuating fast neutrons and absorbing thermal ones^27,28^.

Jing Qiao developed a nuclear shielding material that is both light weight and non-lead by incorporating W and B particles into 6061 Al alloy. Afterwards, the effects of W volume fraction on radiation shielding, mechanical properties, and composite microstructure were investigated. The composites’ ability to shield from γ-rays is improved when the amount of W is increased. Composites of (W/B)Al with a thickness of 2.2 cm are able to absorb 99% of thermal neutrons. Nuclear shielding materials that incorporate structure and function have promising prospects in the (W/B)Al hybrid composites^31^. By employing the mechanical milling process, Hakan Yaykaşlı et al. created a new alloy composition known as Co/Cr/Fe/Ni/Ag, which is an example of a high entropy alloy (HEA). The alloying time was found to have an effect on the crystal size, and the synthesized alloy showed thermal stability over a broad temperature range. A ^137^Cs source and a NaI(Tl) detector system were also used to experimentally determine radiation shielding parameters. The results show that HEAs are feasible and promising concerning the radiation protection applications due to their high radiation shielding properties^32^. The physical properties and nuclear radiation shielding characteristics of four compositions of Al-alloys doped with different weights of Pb were studied by Jamila S. A. et al. The Pb Al-X (X = 1–4) encoding indicates that the samples have a Pb content ranging from 20 to 80%. The gamma transmission experiment, XCOM theory, and Monte Carlo technique are used to study the gamma shielding properties of the alloys that are made. Over the entire chosen energy range, the PbAl-4 alloy consistently ranks as the best shielding material^33^.

Based on all of the above, both aluminum-copper alloy type-2024, Al-2024, and aluminum-manganese alloy type-3003, Al-3003, which have significant contributions in many industries as mentioned above, have been studied concerning their microstructural features and radiation shielding properties against both energetic ionizing photons and fast neutrons.

## Materials and methodology

### Materials

**Figure 1:**
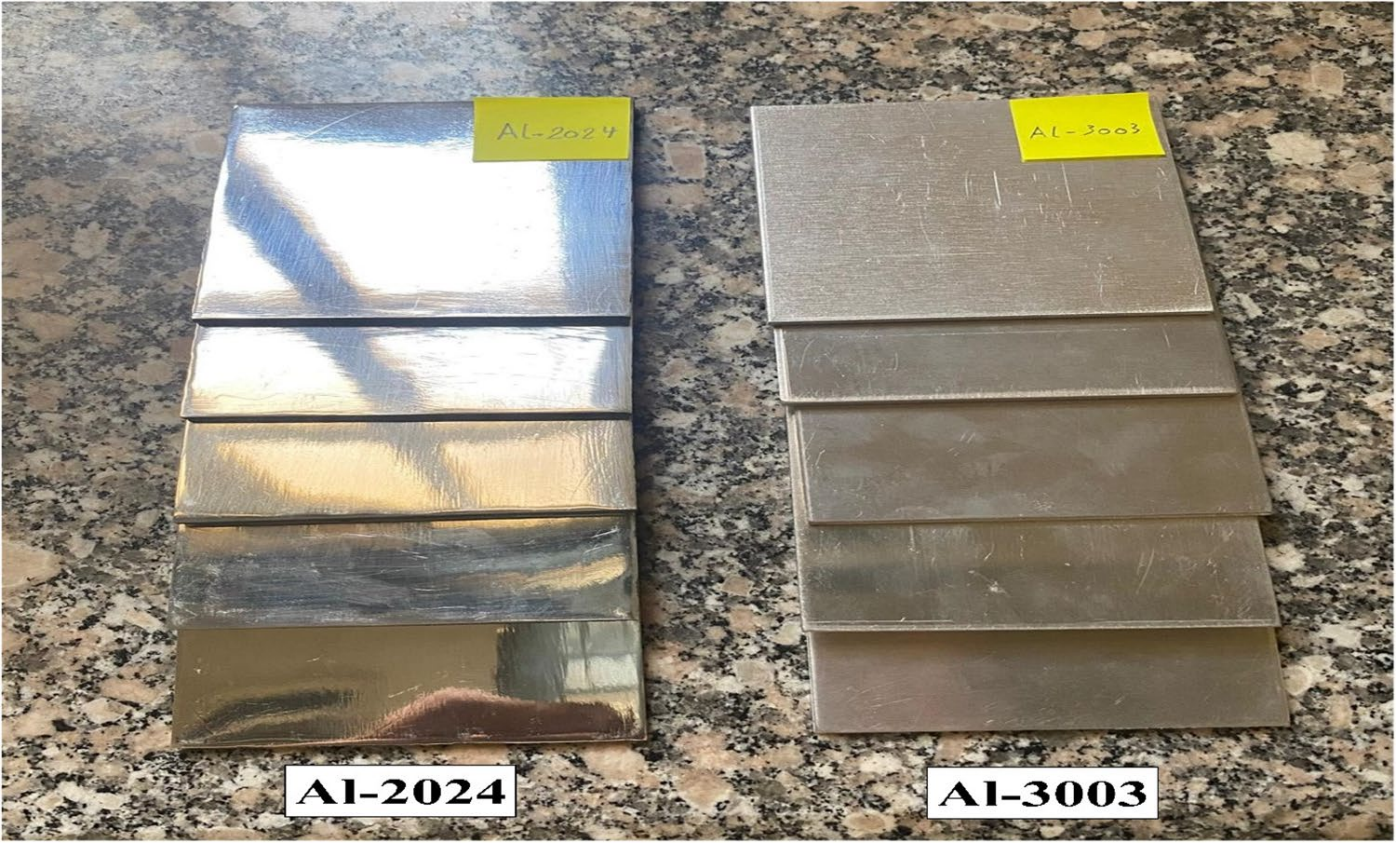
Square shaped sheets of dimensions 3×70×70 mm of Al-Cu type-2024 and Al-Mn type-3003 alloys.

The investigated sheets, with typical chemical compositions shown in Table 1, were received in the form of 3 mm in thickness. They were cut in the form of 70 × 70 mm squares to fit the supporting frame in front of the radioactive sources. They were ground by emery papers with different grades, namely 180, 220, 280, and 320, to normalize and unify the sample’s surface conditions.Then, they were polished by a 1 μm aluminum oxide Al_2_O_3_on a billiard cloth and finally ultrasonically vibrated to remove any solid debris, Fig. 1. Smaller square-shaped samples without any pre-surface treatment were cut for heat treatments and microstructure observation.

**Table 1:** Chemical composition and density of the studied aluminum alloys.

Alloy code	(wt.%)									Density (g/cm^3^)
	Al	Ti	Zn	Cr	Mg	Mn	Cu	Fe	Si	
Al-3003	97.2	0	0	0	0	1	0.5	0.7	0.6	0.5
Al-2024	93.2	0.15	0.25	0.1	1.2	0.3	3.8	0.5	0.5	2.808

### Methodology

Through the use of a scanning electron microscope (SEM) that is accompanied by an energy dispersive X-ray spectroscopy (EDS), the subsequent investigation is broken down into two primary sections. The first section examines the microstructural and localized area elemental analyses for the two aluminum alloys. The second part of the review includes an experimental study on the attenuation of γ-rays, as well as an analytical and theoretical evaluation of both γ-rays and fast neutrons. This evaluation was carried out with the assistance of some reliable software tools, which will be explained in the subsequent sections.

#### Microstructural analysis

Specimens for microstructure observation were cut from the as-received rolled sheets in a direction parallel to the rolling direction and mounted in a plastic mold. The surfaces of these specimens to be studied were prepared according to ASTM E3-11 standard^34^ to reveal the different constituents and their morphologies optically for preliminary investigation and then electronically for higher-level observation. The prepared samples were supported to the holder of the scanning electron microscope (SEM) type ZEISS-EVO MA15 using a double-face stick tape, and a copper strip was used to secure good electrical conductivity. An accelerating voltage of 20 keV and a working distance of about 8 mm were used for SE images and EDS analysis.

The attached secondary electron (SE) detector observed microstructure morphology, whereas the composition variation was obtained using the back-scattered (BS) detector. The energy dispersive X-ray spectroscopy (EDS) detector performed spot, line, and area elemental analysis.

Volume fraction of the second phase particles in all microstructures was calculated by an image analysis software, ImageJ.

#### Radiation shielding investigation

Before illustrating the experimental and theoretical methods used in the current study and clarifying the aim of this part of the study, Table 2 gathers all the shielding parameters of interest required for the assessment, along with the relevant mathematical equations and definitions^35–41^.

**Table 2:** γ-rays and fast neutrons shielding parameters obtained and used for the radiation shielding assessment of the aluminum alloys understudy.

Shielding parameter	Mathematical equation	Definition
Linear attenuation coefficient (µ) in cm^−1^	$\:\mu\:=\frac{\Delta\:\text{l}\text{n}\left(I_x/I_0\right)}{\Delta\:x}$ where I_0_ is the initial beam intensity, I_x_ is the uncollided beam after passing thickness x of the shield.	It is a characteristic shielding parameter that assesses a shield’s capability to attenuate energetic photons, X-rays, and γ-rays.
Fast neutrons removal cross-section (Σ_R_) in cm^−1^	${\textstyle\sum_R}=\sum\limits_1^n\rho_sw_i{\left({\textstyle\sum_R}\rho\right)}_i$ where ρ_s_ and ρ_i_ are respectively, the shield density, and the density of the i^th^ element that constitutes the shield.	It is a characteristic shielding parameter that assesses the capability of a shield to remove fast neutrons from the incident beam.
Half value layer (HVL) in cm	$\:HVL=\frac{ln2}{\mu\:}$	The required shield thickness to attenuate 50% of the coming radiation (energetic photons or neutrons).
Tenth value layer (TVL) in cm	$\:TVL=\frac{ln10}{\mu\:}$	The required shield thickness to attenuate 90% of the coming radiation (energetic photons or neutrons).
Mean free path (MFP) in cm	$\:MFP=\frac1{\mu\:}$	The average distance that can be traveled by the energetic photon in the shield without making any interaction.
Relaxation length (λ) in cm	$\lambda\:=\frac1{\sum_R}$	The average distance that can be traveled by the fast neutron in the shield without making any interaction.
Radiation protection effectiveness (RPE) in %	$RPE=\left(1-\frac{I_x}{I_o}\right)\times100$	Itis an important statistical parameter to take into account when determining the level of attenuation that could be provided by the shield.

#### Experimental study

First, an experimental study was performed using three γ-ray’s sources, Ba-133, Cs-137, and Co-60, to evaluate the actual condition and investigate the concurrence between the experimentally obtained γ-rays shielding parameters and theoretically/analytically obtained ones before giving the green light to further proceed with the theoretical investigation.

Using the abovementioned radioactive sources, γ-rays shielding efficiency of the studied alloys has been experimentally assessed at five energies:0.081, 0.356, 0.662, 1.173, and 1.332 MeV.

**Figure 2:**
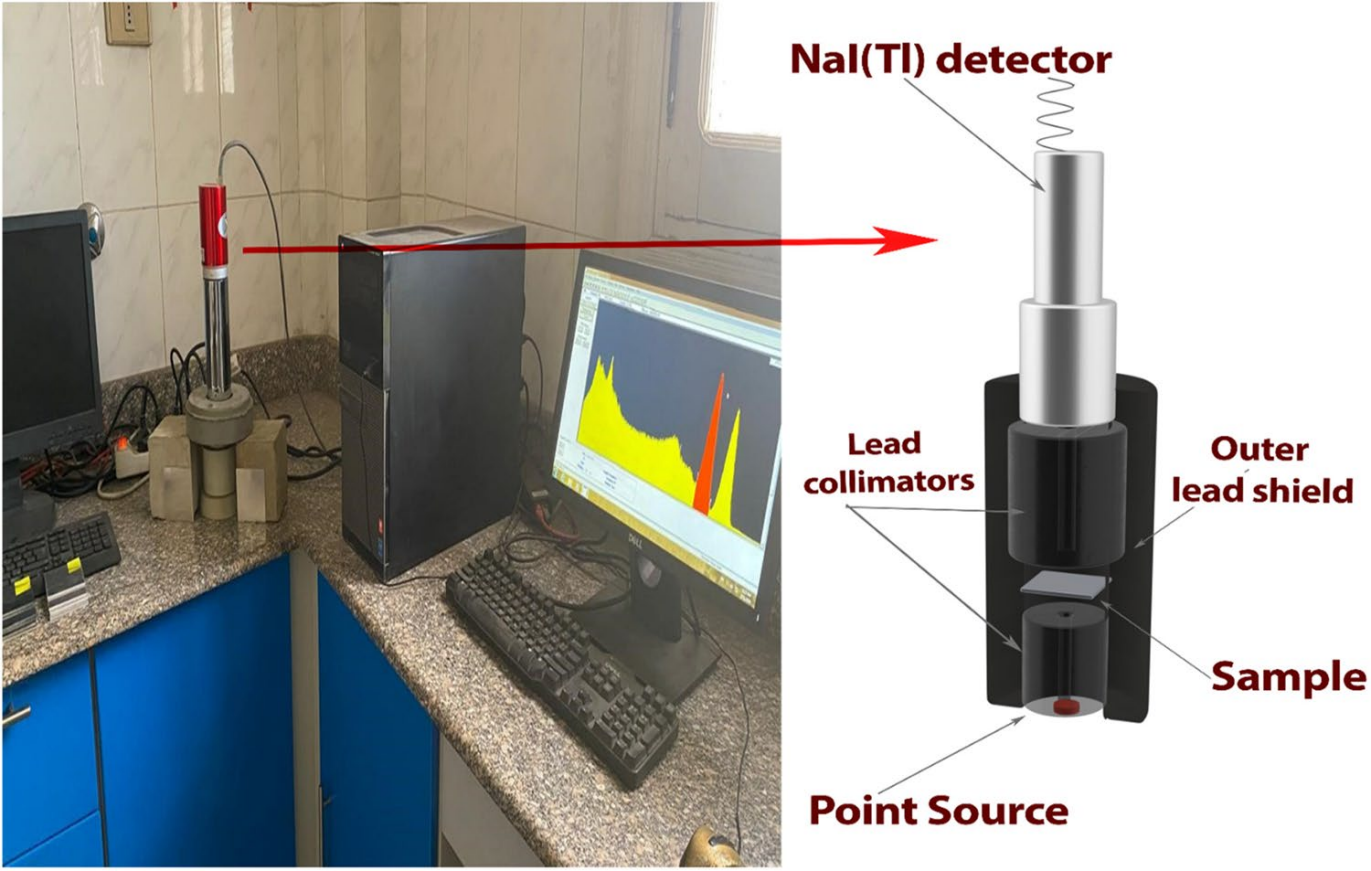
The experimental setup of the γ-rays attenuation measurements.

The experimental testing was performed using a NaI(Tl) 2” × 2” detector coupled with a multichannel analyzer running software (Genni-2000). The γ-rays radioactive source was contained in a 3.5 cm internal cylindrical lead holder with a 3 mm aperture surrounded by an external hollowed “10 cm” lead cylinder (source collimator), and the detector was shielded by cylindrical lead shield (detector collimator) to protect against scattered gamma rays and background radiation and ensure reliable readings^42^. The aluminum alloys in the form of sheets were stacked between the abovementioned collimators, and a straight vertical alignment for all components of the experimental setup was used. All components, and their alignment, of the experimental setup, which are depicted in Fig. 2, are primarily taken into consideration in order to guarantee a narrow beam geometry and significantly reduce the build-up factors, which ultimately results in the acquisition of accurate characteristic shielding parameters for the alloys that are being investigated^43,44^.

It was determined that all the measurements were taken as triplets at each thickness. In order to compile the transmission curves, the intensity of the uncollided γ-ray quanta that have passed through the slab(s) at different thicknesses has been measured and compared to the intensity of the incident beam without any slabs being present (transmission factor). The linear attenuation coefficient (µ) can be determined by plotting a relation between the natural log of the obtained transmission factor and the varied slab(s) thickness. The absolute value of the slope can be taken as the linear attenuation coefficient.

#### Analytical and simulation study

Considering γ-rays shielding theoretical assessment, Phy-X/PSD^45–47^ which is reliable online software that computes the number of essential parameters required to assess the shielding and attenuation capability of the studied material, based only on the material composition and density, is used besides MCNP5^48^.This Monte Carlo simulation code simulates the transit of gamma photons/neutrons through any matter while considering all possible physical interaction mechanisms, depending on an embedded ENDF/B-VII nuclear database^49–51^. Regarding fast neutrons, some reliable software programs and codes, such as; NXCom^51,52^, MRCsC^52^, and MCNP5^48,53^, were found to provide accurate fast neutron shielding assessments^54,55^. However, some differences are found due to the difference in the built-in database used for each program, so the average values have been taken along with putting a specific range where the experimental value should locate within.

**Figure 3:**
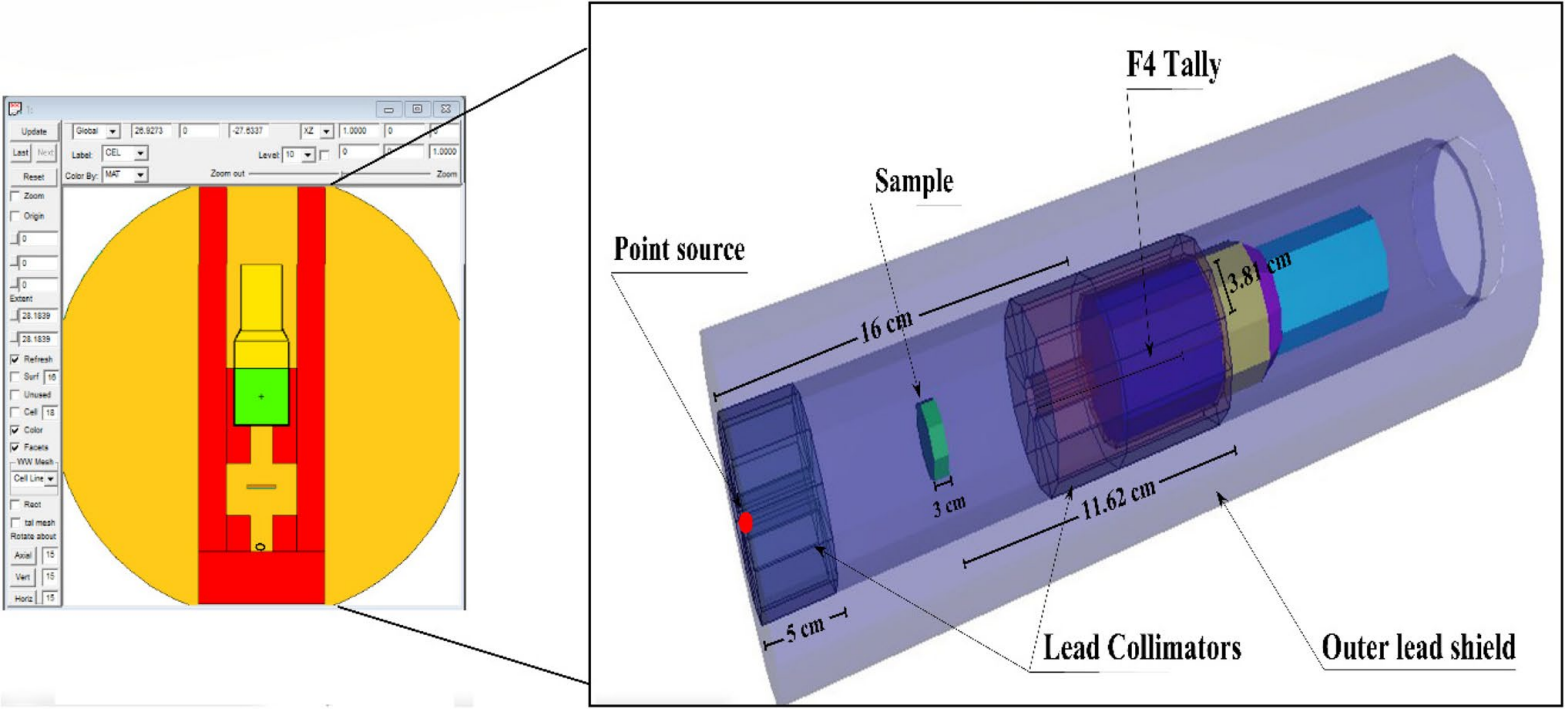
The dynamic view of the radiation attenuation simulation system used for investigating the radiation shielding capabilities of the Al alloys understudy.

The input files for the Monte Carlo simulation depend on the detailed structure of the input file code, which consists of multiple cards (cell, surface, material, tally, etc.), including the experimental setup (e.g., detector dimensions, sample geometry, source height, chemical composition, etc.)^56–58^. The γ-ray shielding set-up was described in the TEXT file in many cell cards (e.g., the γ/n emitting source, lead collimators, Al-alloy sample, and detector. The F4:P tally card was used to determine the track length of the incident γ-photons. The aluminum alloy slabs were created in cylinder geometry. The composition and density of the synthetic Al alloys were made in the material card of the input file. The number of particles emitted from the γ/n sources (NPS) was designed to be more than 10.5E + 6 particles/input file to reduce the random statistical errors to be below 2%^48^. Figure 3 represents the dimensions of the radiation-simulated system used for investigating the Al alloys. To achieve the minimum random statistical errors, all computations use NPS = 11 million particles per run.

## Results and discussion

### Microstructural assessment

**Figure 4:**
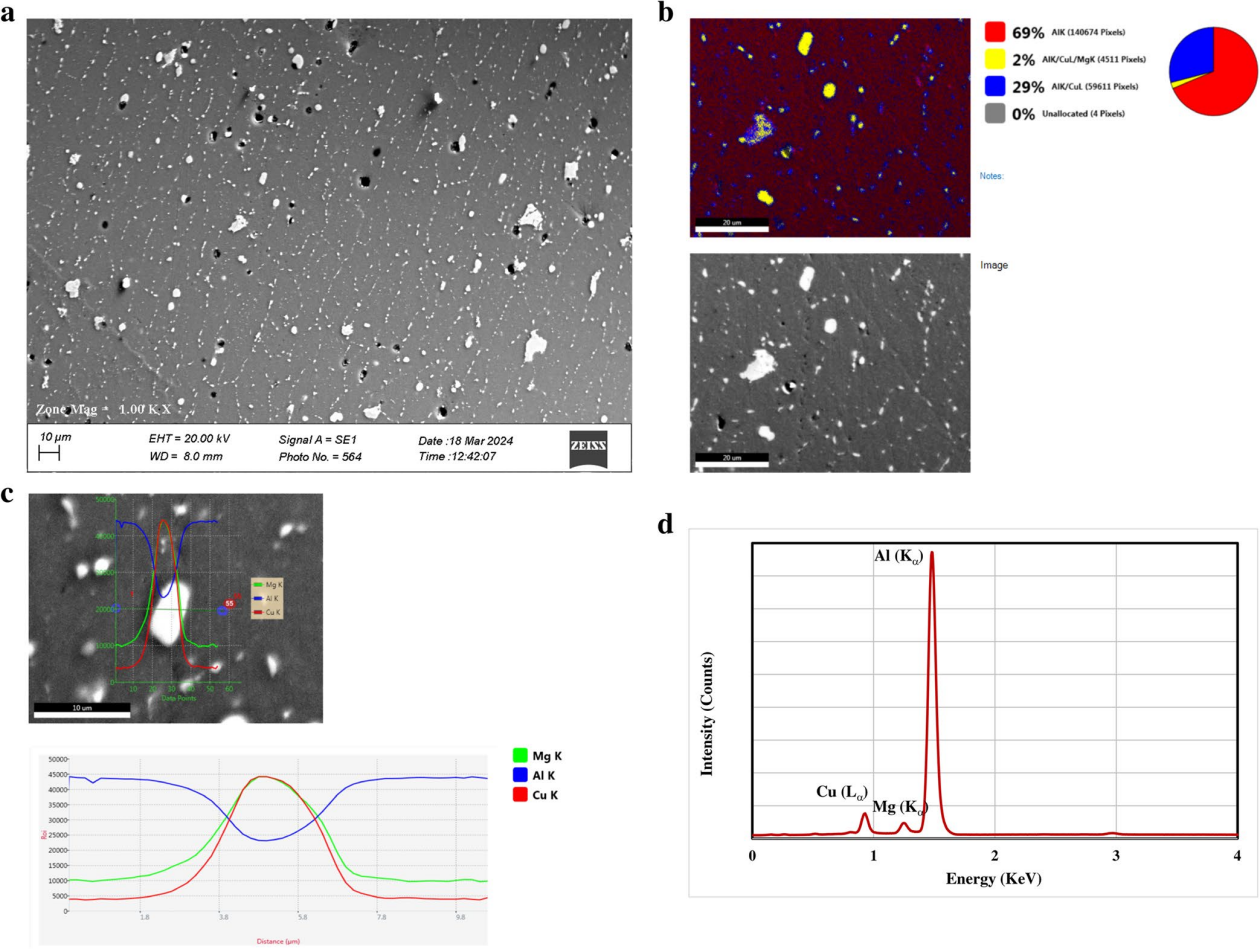
(**a**) SEM microstructure of the aluminum alloy type-2024, (**b**) EDS Elemental mapping of the selected area, (**c**) Line analysis of copper-rich precipitate, and (**d**) Spot analysis of the matrix (Point 1)."Figures 4 and 5 contain poor quality of text inside the artwork. Please do not re-use the file that we have rejected or attempt to increase its resolution and re-save. It is originally poor, therefore, increasing the resolution will not solve the quality problem. We suggest that you provide us the original format. We prefer replacement figures containing vector/editable objects rather than embedded images. Preferred file formats are eps, ai, tiff and pdf.""attached"

Figure 4(a) shows the rolled heat-treatable aluminum alloy type-2024 microstructure. In this figure, coarse second-phase particles, having a size range of 8–15 μm, are aligned in lines parallel to the rolling direction, whereas smaller ones, having a size range of 2–8 μm, decorate the grain boundaries. Volume fraction of these particles was found to have a value of about 7.5%. Black areas and dark holes represent the pull-out large particles separated during preparation because of the weak adhesion with the matrix. This poor adhesion is a result of losing particle coherency with the matrix where the misorientation in-between the atomic planes increases^59^.

Elemental mapping of a selected area is shown in Fig. 4(b) but in the form of phases. Yellow regions coinciding with the course second-phase particles demonstrate the presence of Al/Cu/Mg phases, whereas blue areas overlap the fine precipitates and reveal the Al/Cu phases. The existence of magnesium is proportional to increasing the particle size. Line analysis of coarse precipitate is shown in Fig. 4(c), where a typical bell shape distribution of copper and manganese is observed but with the inverse distribution of Al.

These elemental variations are considered a matter of electron beam interaction with the precipitate and the matrix, not a composition variation within the precipitate itself. Spot analysis of the fine precipitates confirms the disappearance of magnesium and the abundance of copper.

B. Adamczyk-Cieslak et al.^60^ and T. Hashimoto et al.^61^ studied these precipitates in the alloy that was currently being investigated, and they were found to be Al_2_CuMg and Al_2_Cu, respectively. Spot analysis of the alloy matrix presented in Fig. 4(d) shows the existence of these main constituents, Cu and Mg, in solid solution with a Cu percentage up to 2% at room temperature as indicated in the Al-Cu and Al-Mg phase diagrams. It is observed that the Mg percentage in the Al_x_Cu_x_Mg_x_ phase increases with increasing its particle size since the Mg atoms migrate from the matrix toward the particles.

**Figure 5:**
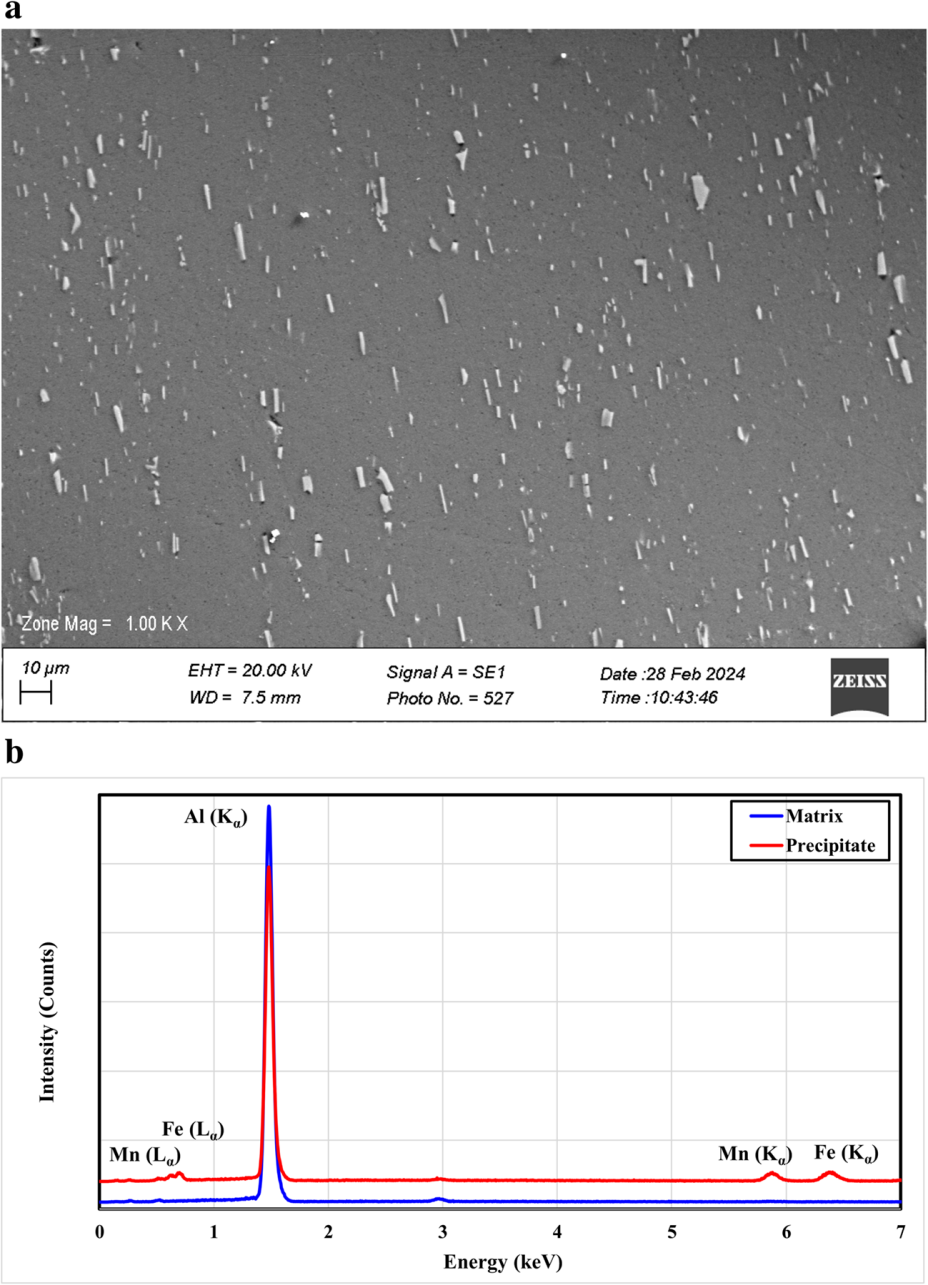
(**a**) SEM microstructure of the as-received aluminum alloy type-3003 and (**b**) EDS spot analysis of matrix and precipitates (point 1 and 2, respectively).

On the other hand, the microstructure of the rolled non-heat treatable aluminum alloy type-3003 is shown in Fig. 5(a). Distributed second-phase particles in the form of a plate-like shape aligned in the rolling direction are observed with particle size relatively smaller than that for the course second-phase particles detected in the case of Al-2024 alloy. In addition, these particles have a lower volume fraction than that found in Al-2024 alloy with a value of 3.5%. Spot analysis of these residues reveals the intense concentration of both Fe and Mn, while the matrix demonstrates the absence of any other constitutional elements except aluminum, Fig. 5(b). This can be easily explained by the relatively low solubility of Fe and Mn in aluminum where they have almost no solubility at room temperature as indicated in the Al-Fe and Al-Mn phase diagrams.

The analyzed second-phase particles were found to match the general chemical formulae, Al_x_Mn and Al_x_(Mn, Fe), as proved in the studies performed on the exact alloy by T. Christopher^62^ and D. Alexander^63^. In the shown microstructure, no pull-out particles came out from their positions as in the former case, which could be attributed to the better adhesion of these second-phase particles to the matrix.

### Radiation shielding assessment

#### γ-rays shielding experimental assessment

**Figure 6:**
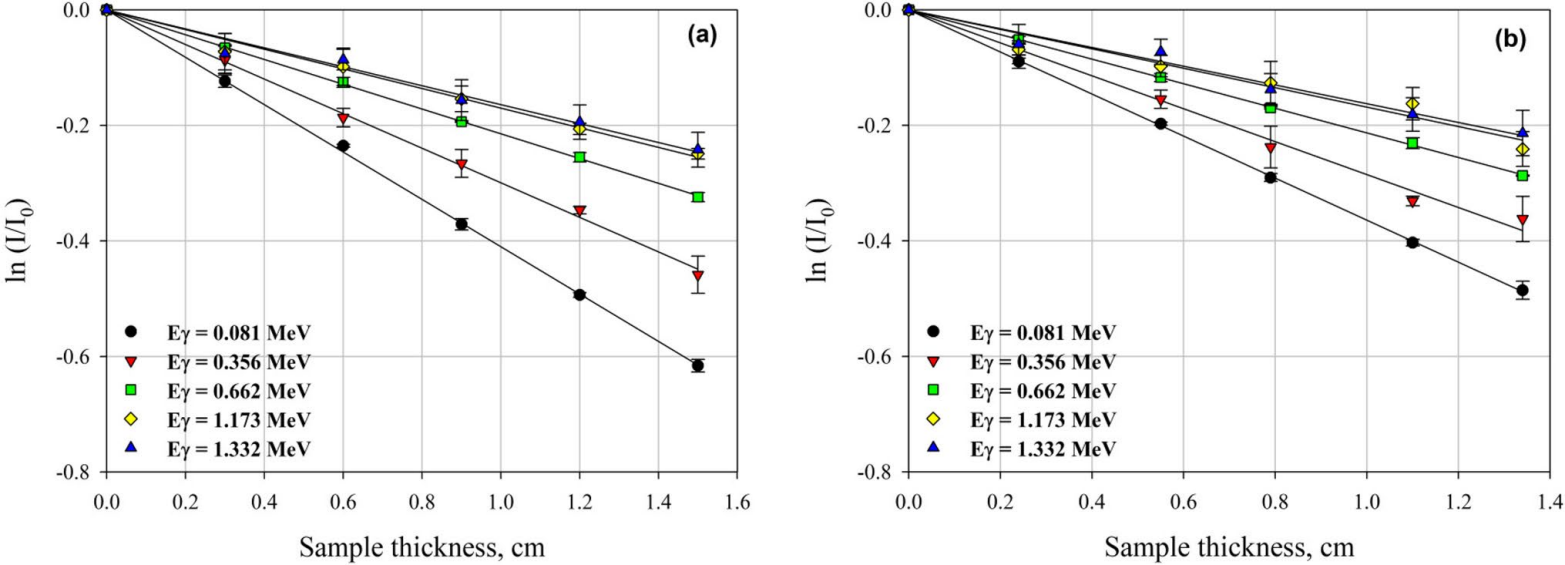
The obtained transmission curves for γ-rays’ attenuation measurements for **a**) Al-2024 and **b**) Al-3003 aluminum alloys.

According to the above-described experimental setup and methodology, the transmission curves have been compiled at the abovementioned five γ-rays’ energies for the studied aluminum alloys, as shown in Fig. 6. The measurements have been performed as triplets, and the absolute value of the slope has been taken as the experimentally obtained linear attenuation coefficient (µ) value of the alloy understudy. Table 3 presents the experimental and corresponding MCNP5 computed µ (cm^−1^) values at the five studied γ-rays’ energies for the investigated aluminum alloys.

**Table 3:** Experimentally obtained and computed linear attenuation coefficients (µ) and the associated percentage differences for the studied Al alloys.

γ-rays’ energy (MeV)	Alloy code	µ (Exp)	µ (MCNP)	%Diff.
0.081	Al-2024	0.4098	0.6519	37.1281
	Al-3003	0.3643	0.5861	37.8518
0.356	Al-2024	0.2990	0.2785	7.3783
	Al-3003	0.2854	0.2697	-5.8171
0.662	Al-2024	0.2150	0.2102	2.2950
	Al-3003	0.2119	0.2043	3.7563
1.173	Al-2024	0.1690	0.1598	5.7720
	Al-3003	0.1684	0.1548	8.7748
1.332	Al-2024	0.1639	0.1495	9.6210
	Al-3003	0.1621	0.1486	9.0740

The experimentally obtained (µ) values for both alloys show the superiority of Al-2024 over Al-3003 alloy at all studied energies. Recalling the SEM and EDS obtained results, the coarse second phase particles, where high-Z elements especially copper are concentrated in, were found having a size range of 8–15 μm and distributed in the main matrix in parallel direction moreover, smaller ones, having a size range of 2–8 μm, were found decorate the grain boundaries and enhance the distribution of these second phase copper rich particles. The volume fraction of these second phase particles comparing to the main aluminum matrix was found about 7.5%.

On the other side, considering Al-3003 alloy, the second phase particles were found taking a plate-like shape aligned in the rolling direction with particle size notably smaller than that for the course second-phase particles detected in the case of Al-2024 alloy moreover, these second phase particles are composed of Fe and Mn, which means lesser high-Z elements content comparing to the case with the Al-2024 alloy, with a volume fraction equals only about 3.5%.

All the former explanations, enforce and support the γ-rays shielding superiority of Al-2024 alloy over the Al-3003 at all experimentally investigated γ-rays’ energies especially at Eγ = 0.081 MeV as the dominant mechanism at this energy is the photoelectric mechanism that has a cross section positively correlates with ≈ Z^4.5^. Thus, any increase in the high-Z elements concentrations especially in the distributed second phase particles would have significant positive effect on the γ-rays shielding capability of this alloy.

On the other hand, this superiority has been found decreasing with increasing the incident γ-rays’ energy as the available time for photons’ interactions with the shield constituents decreases. Also the dependency of the significant interaction mechanism “Compton scattering” which is the dominant mechanism at the studied high energy range on both atomic number and density of the shield is the least^64,65^. Another important prediction based on the observed dark holes which represent the pull-out large particles separated during preparation of the Al-2024 alloy, is that the γ-rays shielding superiority of this alloy over the other studied Al-3003 alloy would be greater if the adhesion between the second phase particles and the main aluminum matrix can be enhanced.

Based on the experimentally obtained and the computed linear attenuation coefficients (µ) along with the associated percentage differences, a good agreement has been observed at all studied energies except at the lowest energy, 0.081 MeV, which could be attributed to the great sensitivity at lower photons’ energies to any variation in the atomic number, constituents’ contents, homogeneity, and distribution of the second phases in the main alloy matrix, due to the dominancy of the photoelectric mechanism at this low energy. Heterogeneity attributed to the distribution of the relatively high-Z second phase particles in the main Al matrix even if it was found not so significant, still can cause the notable difference between the computed value and the experimentally obtained one specifically at this low energy which again dominated by the sensitive photoelectric photon/matter interaction mechanism unlike the case with the other studied energies which are controlled by the less-sensitive Compton scattering mechanism.

Beside the abovementioned explanation for why the notable difference between the experimental and the computed (µ) value at photon energy 0.081 MeV, it must be declared that most of software tools used for radiation shielding assessment deal with the composite shield as if it is homogenized, ignoring the internal form of distribution and constituents’ particle size and relying only on the constituents’ weight fractions and the overall shield density^19^. In contrast, the agreement between the experimentally obtained and the computed linear attenuation coefficients (µ) at Eγ = 0.662 MeV is the highest because at intermediate gamma rays’ energies, the dominant interacting mechanism is Compton scattering, which is the interaction mode that depends the least on the atomic number of the shield so, any possible; fluctuation, variability, or slight heterogeneity, will cause lesser differences between the experimental and computed values unlike the case with lower energies as explained earlier^19,66^. Thus, to obtain reliable results and credible characterizing values for the accurate shielding parameters for the truly prepared alloys considered in the current study, the analytical/theoretical γ-rays’ shielding assessment has been extended to range from 0.1 MeV to 2 MeV only.

#### γ-rays shielding analytical assessment

Before investigating the computed values of the various γ-rays’ shielding parameters, a comparison between the calculated (µ) values within the investigated energy range using both Phy-X/PSD software45 and the model created by MCNP5^48^ has been performed, as clarified in Table 4, to do further verification before proceeding solely using the Monte Carlo model.

**Table 4:** Computed linear attenuation coefficients (μ) using Phy-X/PSD software and the created MCNP5 model, along with the associated percentage differences for the studied samples.

Energy, (MeV)	Linear attenuation, µ (cm^−1^)								
	Al-3003			Al-2024			Al-Pure		
	Phy-X	MCNP	%Diff.	Phy-X	MCNP	%Diff.	Phy-X	MCNP	%Diff.
0.1000	0.4762	0.4879	2.3972	0.5168	0.5280	2.1306	0.4762	0.4879	2.3972
0.2000	0.3342	0.3424	2.3733	0.3480	0.3555	2.1094	0.3342	0.3424	2.3733
0.3000	0.2839	0.2886	1.6086	0.2938	0.2980	1.4297	0.2839	0.2886	1.6086
0.4000	0.2525	0.2562	1.4487	0.2608	0.2642	1.2876	0.2525	0.2562	1.4487
0.5000	0.2298	0.2327	1.2696	0.2371	0.2398	1.1284	0.2298	0.2327	1.2696
0.6000	0.2122	0.2146	1.1179	0.2189	0.2211	0.9936	0.2122	0.2146	1.1179
0.8000	0.1860	0.1873	0.6705	0.1919	0.1930	0.5960	0.1860	0.1873	0.6705
1.0000	0.1671	0.1681	0.6100	0.1723	0.1733	0.5422	0.1671	0.1681	0.6100
2.0000	0.1176	0.1181	0.3799	0.1213	0.1217	0.3377	0.1176	0.1181	0.3799

The simulated µ values at all studied energies are in excellent agreement with those calculated by the Phy-X program, with a maximum %Diff. hasn’t reached 2.4% considering all the investigated aluminum-based samples.

**Figure 7:**
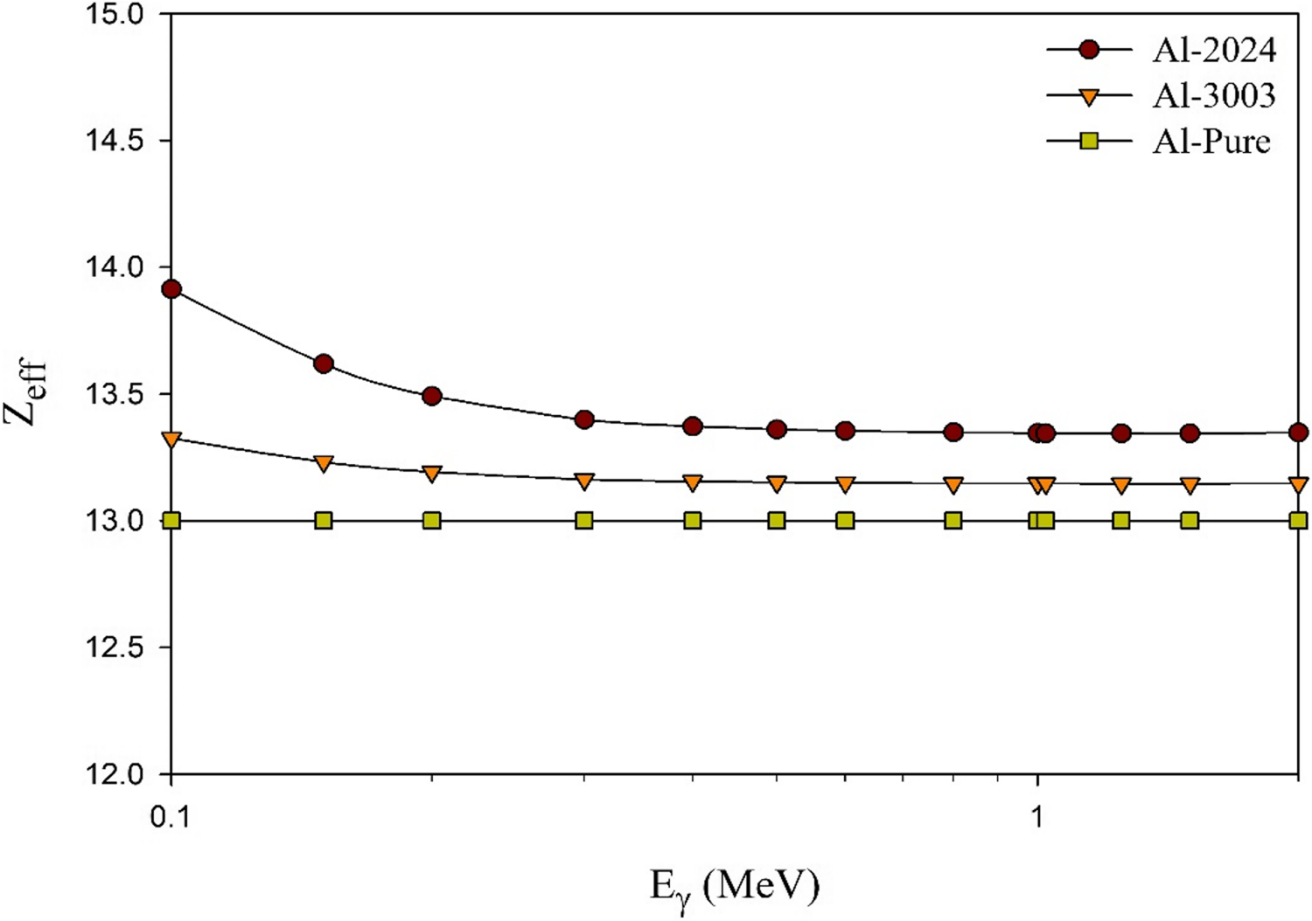
The computed effective atomic numbers with photon energy for the studied aluminum alloys in comparing to pure aluminum.

**Figure 8:**
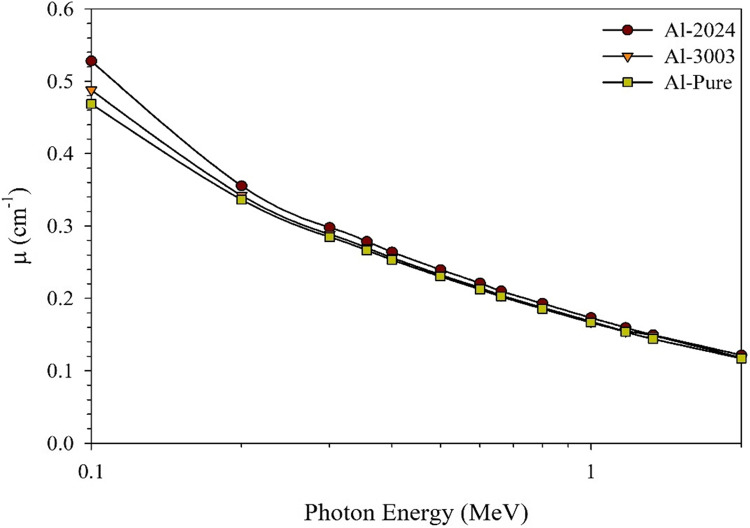
The computed linear attenuation coefficients using MCNP model for the studied aluminum samples.

Considering the effective atomic numbers (Z_eff_) and µ values computed for the studied aluminum samples within the energy range of interest, Figs. 7 and 8 depict the interrelation between both shielding parameters.

Based on the results shown in the previous two figures, Z_eff_ is known to be attributed to the γ-rays interaction modes with the attenuating medium thus, its value usually varies with the photon energy^67,68^. As a consequence, higher values are observed at low energies as a result of the control of the photoelectric mechanism, which significantly depends on the atomic numbers of the shield constituents. On the other hand, for the current energy range that was studied, which ranged from 0.1 MeV to 2 MeV, the lowest Z_eff_ values were observed throughout the majority of this range, with the exception of the onset, moreover, the observed values were almost independent of the incident photon energy. This can be attributed to the dominancy of the Compton scattering mechanism at these energies. Al-2024 alloy possesses the highest values, relatively, especially at the start of the studied energy range, as high-Z alloying elements such as copper have a non-negligible percentage, 3.8%, in this aluminum alloy in contrast to pure aluminum and the other Al-3003 alloy that has small percentages of high-Z alloying elements like copper and iron.

The calculated (µ) values comply with the obtained Z_eff_ values. Again, a smooth decrease, not a sharp one, has been observed for all studied samples with the photon energy except at the beginning of the curves where the photoelectric effect is still significant in defining the way of interaction between the incident photon and the attenuating medium. The remaining of the studied energy range is dominated by the Compton scattering photon/matter interaction mechanism, which is the mechanism that shows the minor dependency on the shield effective atomic number, so even if Al-2024 alloy is still in the lead, the differences between the three studied aluminum samples become smaller stepping toward the end of the investigated energy range.

**Figure 9:**
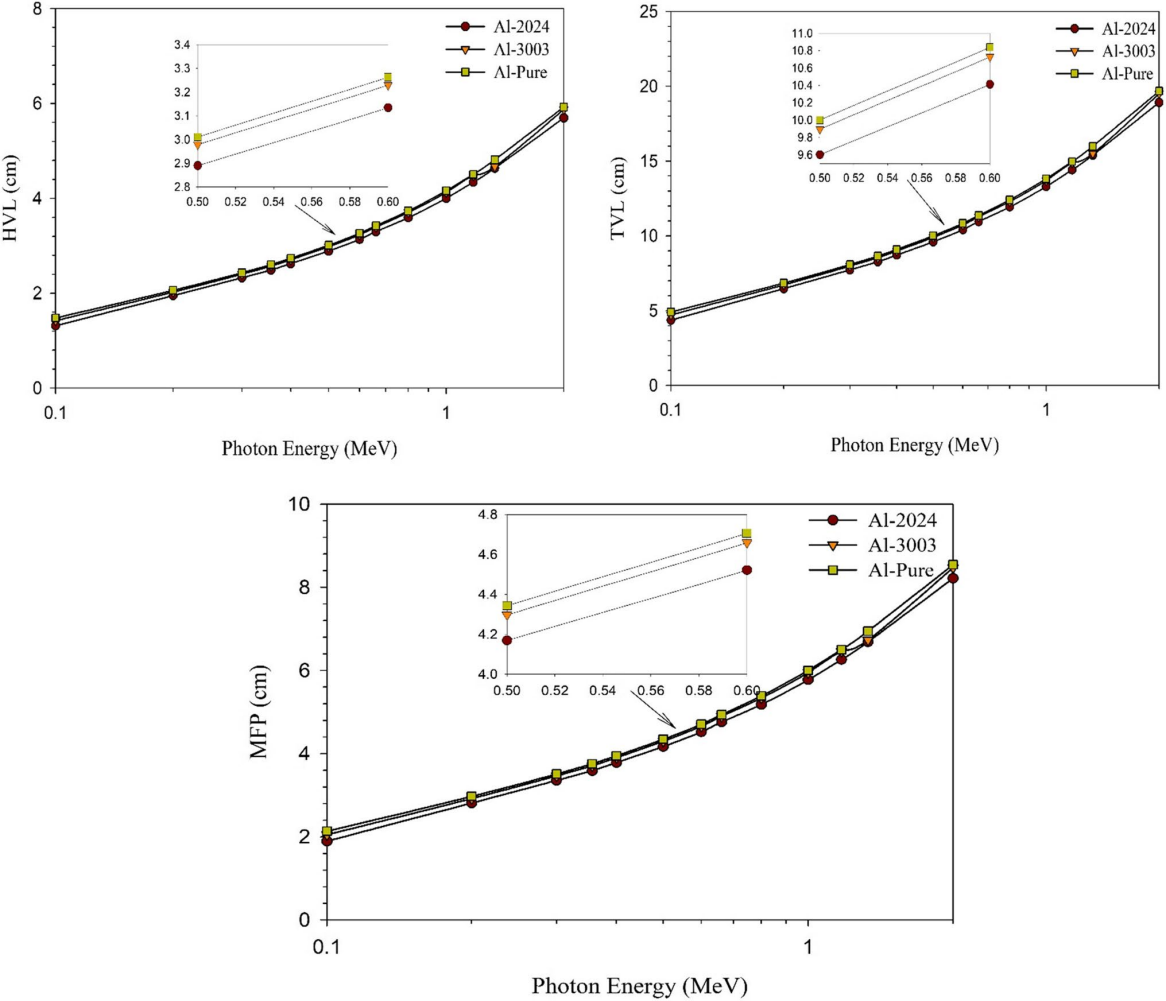
The half value layer (HVL), tenth value layer (TVL), and mean free path (MFP) for pure aluminum and the other prepared Al alloys vs. the photon energy.

The required shield thicknesses to attenuate; 50%, 90%, and about 67%, of the incident photons, i.e., HVL, TVL, and MFP, respectively^37,69^, are presented in Fig. 9 for the studied aluminum samples.

All shielding thicknesses increase as photon energy increases for all aluminum samples due to the increase in the photon escaping probability and the decrease in the attenuation cross section denoted by the linear attenuation coefficient (µ), as illustrated above. For all studied energies, Al-2024 alloy possesses, relatively, the smallest required shielding thicknesses for the studied three shielding parameters. The differences are relatively small as the alloying elements, especially those with high-Z, are with small percentages compared to the main aluminum matrix.

As mentioned before, RPE is an essential statistical parameter that should be taken into account when determining the level of attenuation that the shield should provide^54,70^.

**Figure 10:**
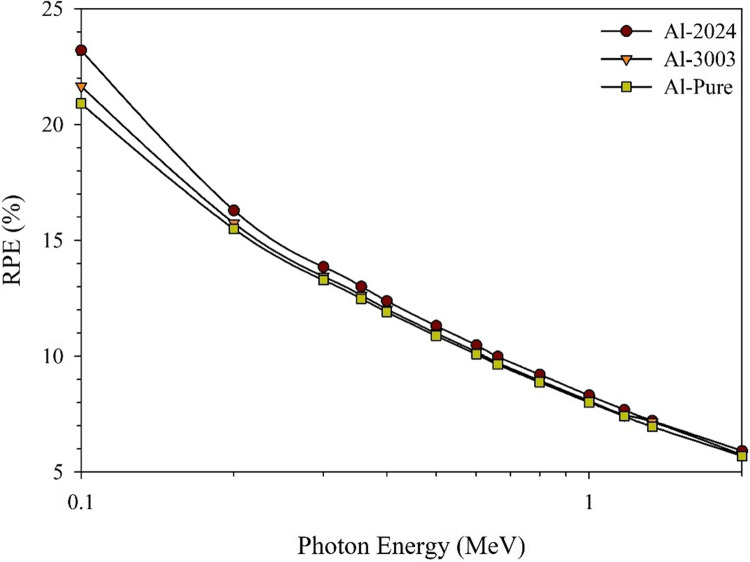
The computed radiation protection efficiency, RPE, at selective thickness equals 0.5 cm for the studied aluminum samples.

Figure 10 shows that the RPE values are more than 20% at low γ-rays’ energies (around 0.1 MeV). When the incident photon energy increases, the penetration power of the incident photons also increases, leading to a significant decrease in the RPE (%) levels^71,72^. Therefore, at the start of the studied energy range, the superiority of the Al-2024 alloy over the other samples, thus, its γ-rays’ shielding efficiency, can be considered tangible and effective while dealing with low γ-rays’ or traditional X-rays radiation fields. The RPE values dropped from about 23.2, 21.6, and 20.8% at 0.100 MeV to only 5.7, 5.9, and 5.6% at E_γ_ = 2 MeV, for the studied samples; Al-2024, Al-3003, and Al-Pure, respectively.

#### Fast neutrons shielding analytical assessment

**Figure 11:**
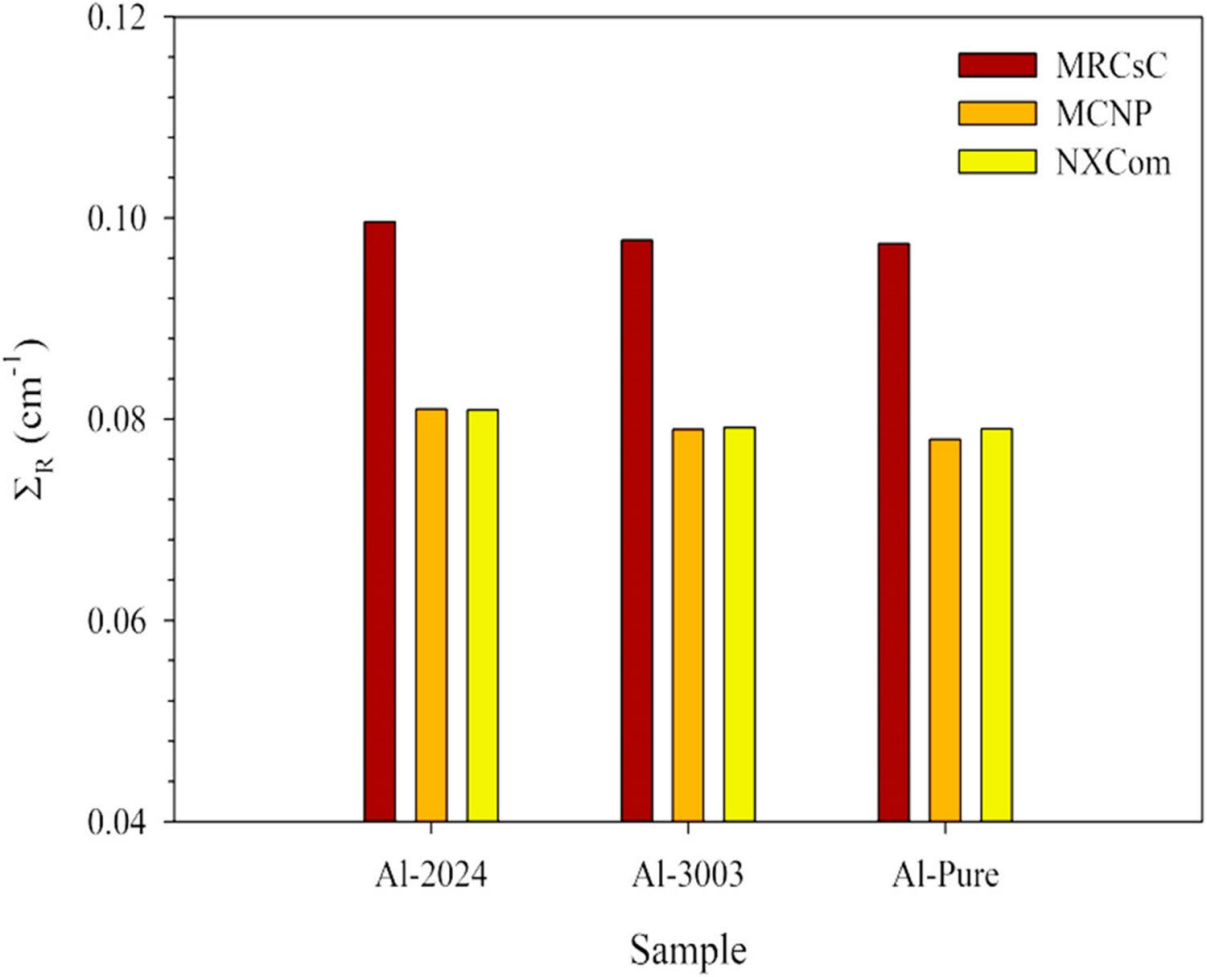
The computed fast removal cross sections for the studied aluminum samples.

**Figure 12:**
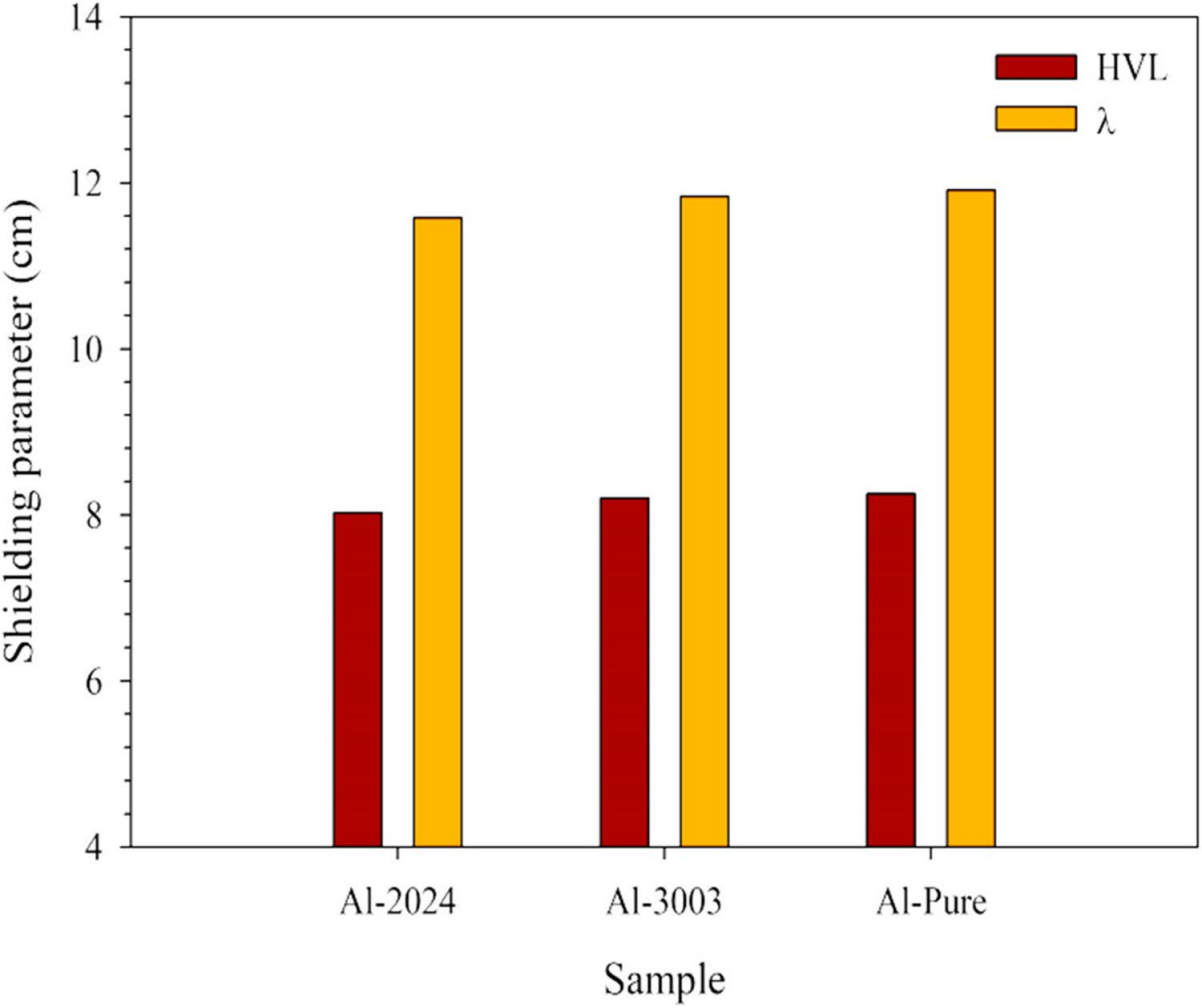
The computed fast removal cross sections for the studied aluminum samples.

Considering shielding against fast neutrons, NXCom^55^, MRCsC^52^, and MCNP5^48,73,74^ software tools were used for calculating the macroscopic fast neutrons removal cross sections of the aluminum samples understudy. The used software tools rely mainly on the studied shield composition and measured density and don’t consider other microstructural details like the heterogeneity of the shield and second phase particle size. However, these tools still can provide sufficient preliminary estimation for fast-neutrons shielding properties of the investigated alloys especially in the absence of the capability to conduct an experimental study. The same trend can be observed for all samples, as shown in Figs. 11 and 12, putting Al-2024 again in the first place with an average Σ_R_ equals 0.0872 cm^−1^ and corresponding average HVL and λ equal 8.026 and 11.579 cm, respectively. In contrast, pure aluminum has the most negligible Σ_R_ value (0.0845 cm^−1^) and the greatest thicknesses for both HVL and λ, equal to 8.254 and 11.909 cm, respectively. The differences that have been captured regarding the results obtained via the three tools are logic as each one of them uses a different version of a built-in database such as ENDF/B-VII^75^ that is employed by the MCNP program, and the latest version ENDF/B-VIII^76^ that MRCsC program relies on while computing the parameter. However, utilizing the obtained results to set the margin where the accurate experimental value should be located within is something verified in previously performed researches^77–79^.

Possessing the highest density and relative content of high-Z elements like copper can be considered the reason for putting Al-2024 alloy in the lead regarding attenuating fast neutrons that could be achieved by increasing neutrons/alloy interactions depending mainly on the inelastic scattering mechanism.

## Conclusions

- The relatively higher weight% of alloying elements in Al-Cu alloy type-2024 than that in Al-Mn alloy type-3003 forms a higher volume fraction of second-phase particles in the former alloy.
- In the heat-treatable aluminum alloy type-2024, second-phase particles of Al_2_Cu and Al_2_CuMg were revealed with a decorative pattern around the grain boundaries, while in the non-heat-treatable aluminum alloy type-3003, different forms of Al_x_Mn and Al_x_(Mn, Fe) with regular plate-like shapes were aligned in the rolling direction.
- Adhesion between the matrix and second phase particles in the aluminum alloy type-2024 was weaker than in the aluminum alloy type-3003, where black holes representing pull-out particles were observed in the former alloy and could decrease the radiation shielding capacity of the alloy.
- The heat-treatable aluminum alloy type-2024 possessed the highest γ-rays’ shielding parameters compared to the non-heat-treatable aluminum alloy type-3003 and pure aluminum.
- The superiority of Al-2024 alloy in shielding against energetic ionizing photons was appreciated for low-energy γ-rays, indicating the feasibility of using this alloy as a shield against X-ray radiation fields.
- Homogeneity of the alloy beside increasing the high-Z second phase content can increase the shielding capability especially against X-rays and γ-rays.
- Considering attenuation capabilities against fast neutrons, Al-2024 alloy was the best but with a slight degree of superiority above the other studied alloy.

## Data Availability

All data generated or analyzed during this study are included in this published article.
